# Supplementary material for: Association of Sentinel Node Biopsy and Pathological Report Completeness with Survival Benefit for Cutaneous Melanoma and Factors Influencing Their Different Uses in European Populations
Source: Cancers (Basel). 2022 Sep 8;14(18):4379. doi: 10.3390/cancers14184379 (PMC9497177; doi:10.3390/cancers14184379)
Supplement: Supplementary file 1 [file cancers-14-04379-s001.zip › cancers-1828974-supplementary.pdf]

Supplementary Table S1 - Total number of cutaneous melanoma cases submitted for inclusion in the HR study by country and registry, with criteria for selection of cases.

Country	Cancer Registry	Number of cases	Criteria for selection
Bulgaria	Bulgaria National	319	All incident cases in the Sofia-city administrative district for the period 2011-2013
Estonia	Estonia National	381	All incident cases in 2010-2011
Italy	Modena	231	All incident cases in 2011-2012
Italy	Ragusa	136	All incident cases in 2009-2012
Italy	Veneto	549	All incident cases in 2013
Portugal	Northern Portugal	300	All patients consecutively admitted in the unique specialized oncology center existent in the cancer in the registry coverage area, until the minimum number of cases requested by the HR study was achieved
Portugal	Southern Portugal	470	All incident cases in 2012
Spain	Girona	287	All incident cases in the province of Girona n 2009-2012
Spain	Granada	341	All incident cases in 2011
Spain	Navarra	306	All incident cases in the region in the year 2013, plus in January and February 2014 in order to reach the requested number of cases
Switzerland	Geneva	473	All incident cases in 2011-2013

Supplementary Table S2. Number of cases and adjusted 5-year Relative Excess rate of Risk of death (RER) with 95% Confidence Interval (CI) for cutaneous melanoma patients diagnosed in 2009-2013 in four European countries.

	N	*RER*	*95% CI*	*p-value*
**Country[2]**				
Estonia	345	**1.01**	[0.72-1.41]	0.960
Italy	355	**1.21**	[0.83-1.76]	0.314
Portugal	300	**0.96**	[0.70-1.31]	0.787
Spain	922	**0.85**	[0.66-1.10]	0.229
**Age at diagnosis (years)**				
15-54	759	**0.82**	[0.52-1.28]	0.383
55-64	345	**0.98**	[0.60-1.60]	0.942
65-74	376	**1.02**	[0.66-1.58]	0.942
75+	442	**1**		
**Sex**				
Men	886	**1**		
Women	1036	**0.86**	[0.62-1.19]	0.371
**Receiving a complete PR8[1]**				
Yes	440	**1**		
No	1482	**2.38**	[1.41-4.02]	0.001
**Mitotic index**				
<1_per_mm²	533	**1**		
≥1_per_mm²	841	**3.10**	[1.03-9.38]	0.045
Not mentioned in PR	548	**2.01**	[0.64-6.29]	0.229
**Thickness**				
<= 1mm	969	**0.12**	[0.05-0.27]	0.000
1.01mm-2mm	301	**0.20**	[0.11-0.40]	0.000
2.01mm-4mm	250	**0.63**	[0.43-0.92]	0.017
> 4mm	347	**1**		
Unknown	55	**0.45**	[0.13-1.51]	0.195
**Ulceration**				
Absent	477	**1**		
Present	1337	**2.11**	[1.43-3.13]	0.000
Not mentioned in PR	108	**1.72**	[0.57-5.12]	0.333
**Nodal stage**				
N0	1324	**0.74**	[0.48-1.13]	0.162
N1	37	**2.43**	[1.37-4.34]	0.003
N2	17	**1.19**	[0.53-2.66]	0.667
N3	16	**1.57**	[0.68-3.64]	0.288
N+	3	**2.98**	[0.63-14.03]	0.168
Nx	525	**1**		
**M stage**				
M0	1598	**0.69**	[0.37-1.30]	0.252
M1	44	**5.35**	[2.57-11.12]	0.000
Mx	280	**1**		

**Charlson comorbidity index (CCI)**				
CCI 0	1275	**1**		
CCI >=1	566	**1.51**	[1.09-2.09]	0.014
Unknown	81	**0.84**	[0.28-2.51]	0.745

Notes:
[1] Eight recommended histopathological items: Breslow thickness, ulceration, histological subtype, mitotic rate, growth phase, lymphocyte infiltration, tumor regression, and vascular or neural involvement.
[2] Reference for country is the European mean.
